# Prevalence of toxigenic fungi in common medicinal herbs and spices in India

**DOI:** 10.1007/s13205-016-0476-9

**Published:** 2016-08-05

**Authors:** Visenuo Aiko, Alka Mehta

**Affiliations:** School of Bio Sciences and Technology, VIT University, Vellore, 632014 India

**Keywords:** Aflatoxin B_1_, Citrinin, Fungal load, Mycotoxins

## Abstract

Mycotoxins are unavoidable contaminants of food grains, feeds, medicinal herbs, and spices, posing as health threat to animals and humans. The objective of this study was to screen medicinal herbs and spices for fungi and mycotoxin contamination and evaluate their safety. Sixty-three samples were examined for fungal contamination and fungal load determined using standard microbiological method. Aflatoxin and citrinin were detected using thin layer chromatography and high-performance chromatography technique. Fifty-eight out of the 63 samples were contaminated, while five were free from fungal contamination. Analysis revealed that 47 % of the samples had a fungal load above 1 × 10^3^ cfu/g which is the permissible limit set by World Health Organization. The samples *Mesua ferrea*-II and *Terminalia chebula*-III had the highest fungal load, i.e., 5.0 × 10^4^ cfu/g. A total of 187 fungi were isolated, out of which 28 were toxigenic which included 19 aflatoxin-producing *Aspergillus flavus* and 9 citrinin-producing *Penicillium citrinum*. The natural contamination with aflatoxin B_1_ was detected only in one sample, i.e., *Arachis hypogaea* (groundnut) which was present beyond the permissible limit. Though toxigenic fungi were isolated, mycotoxins were not detected from any of the medicinal herbs and spices. Medicinal herbs and spices are susceptible to toxigenic fungi; however, they also possess intrinsic factors that inhibit mycotoxin contamination. This study provides a basis in assessing the degree of fungal and potential mycotoxin contamination in medicinal herbs and spices.

**Electronic supplementary material:**

The online version of this article (doi:10.1007/s13205-016-0476-9) contains supplementary material, which is available to authorized users.

## Introduction

Mycotoxins which literally mean “fungus poison” are secondary metabolites produced by saprophytic fungi, such as *Aspergillus flavus*, *A. ochraceus*, *Fusarium moniliforme*, *F. graminearum*, *Penicillium citrinum*, *P. expansum*, etc. The important mycotoxins causing significant health hazards are aflatoxins, ochratoxin A, citrinin, *Fusarium* toxins, patulin, and zearalenone. They are toxic to both animal species and humans and are reported to be carcinogenic, hepatotoxic, nephrotoxic, cytotoxic, mutagenic, and immuno-suppressive (Hussain and Brasel 2001).

Mycotoxins occur as natural and unavoidable contaminants on a variety of food commodities posing as potential health hazard. The occurrence of fungi and mycotoxins in medicinal herbs has been reported from around the world. Studies in India showed the natural occurrence of aflatoxin B_1_, citrinin, ochratoxin A, and zearalenone in medicinal plants and herbal drugs, such as *Asparagus racemosus*, *Cinnamomum zeylanicum*, *Elettaria cardomomum*, *Piper nigrum*, *Zingiber officinale*, etc. (Roy et al. 1988; Chourasia 1995; Thirumala-Devi et al. 2001). The incidence of toxigenic fungi producing aflatoxins, ochratoxin A, and fumonisin on medicinal herbs was reported from Argentina (Rizzo et al. 2004). An investigation from South Africa showed the presence of fumonisin B_1_ in dietary and medicinal wild plants (Sewram et al. 2006). Bugno et al. (2006) had reported the occurrence of aflatoxins-, ochratoxin A-, and citrinin- producing *Aspergillus* and *Penicillium* in medicinal herbs in Brazil. In Saudi Arabia, the presence of aflatoxin B_1_ (12–40 µg/kg) in *Pimpenella anisum*, *P. nigrum*, *Mentha piperita*, and *Origamun majorana* was reported (Bokhari 2007). A survey in Korea on spices and processed spice products for aflatoxin contamination showed the presence of aflatoxins at 0.08–4.66 µg/kg level in samples of red pepper and ginger products (Cho et al. 2008). Multicontamination of mycotoxins with T-2 toxin, zearalenone, aflatoxins, ochratoxin A, deoxynivalenol, citrinin, and fumonisin were detected in 84 medicinal herbs surveyed in Spain (Santos et al. 2009).

In India, the medicinal properties of plants have been greatly exploited in Ayurveda which is one of the traditional systems of Indian medicine. Herbs are used as dietary and health supplements and the demand for medicinal herbs as an alternative medicine has increased over the last few years. These herbs are often prone to fungal and mycotoxin contamination; the efficacy and safety of medicinal herbs are questionable, and the detailed reports on the same are still scarce (Calixto 2000; Trucksess and Scott 2008). The consumption of medicinal herbs contaminated with mycotoxins may cause ill effects rather than improving the well-being of an individual.

The objective of this study was to evaluate the safety of medicinal herbs and spices by screening for fungal and mycotoxin contamination. In addition, the fungal load in the samples was determined, and toxigenic fungi were identified.

## Materials and methods

### Sampling

Medicinal herbs and spices were collected from retail market and herbal farm in polythene bags which were then brought to the laboratory for testing. The samples obtained from the herbal farm have been certified by CSIR-NISCAIR-National Institute of Science Communication and Information Resources, New Delhi. The samples obtained from the market have been confirmed with the Handbook on Herbaria in India and Neighboring Countries (Viswanathan et al. 2000).

A total of 63 samples which includes 38 different types of commonly used herbs, herbal products, spices, and food materials were analyzed for fungal and mycotoxin contamination. Samples were selected based on the common usage and regular availability in retail shops. The samples were recollected after a period of 6 and 12 months based on their availability and analyzed within a week of collection and then stored in a cold room for future use.

The moisture content of the samples was checked by drying at 80 °C in a hot air oven until their weight remained constant. The total moisture content was then calculated using the given formula (Nielsen 2010):

$$ {\text{Moisture }}\;\% \, \; = \; \, \left( {{\text{A }}\;{-}\;{\text{ B }}\; \div \;{\text{ A}}} \right) \, \; \times \, \; 100, $$

where A is the wet weight and B is the dried weight of samples.

## Materials

Culture mediums used were Rose Bengal Chloramphenicol Agar (RBCA), Potato Dextrose Agar (PDA), and Yeast Extract Sucrose broth (YES) from HiMedia, India. Thin layer chromatography plates (TLC Silica gel 60 F_254_) were procured from Merck (Darmstadt, Germany), aflatoxin mix (B_1_, B_2_, G_1_, and G_2_) from Supelco (Bellefonte, PA, USA) and citrinin from Sigma (St. Louis, MO, USA).

### Screening samples for fungal contamination and enumeration of fungal load

One gram of powdered sample was diluted in 10 ml sterile 0.1 % aqueous peptone. 200 µL of this suspension (dilution factor 10^−1^) was inoculated onto PDA and RBCA by the spread plate method (Hocking et al. 2006). Direct plating was also performed by placing small pieces of unsterilized samples on the medium without dilution. The plates were incubated at 28 °C for 2–7 days. The fungal colonies were counted and the total fungal load was calculated as colony forming unit (CFU) per gram of the sample. The different fungal isolates were subcultured on PDA till pure cultures were obtained. The isolated fungi were identified macroscopically and microscopically by their cultural and morphological characteristics. These pure cultures were further analyzed for toxin production and transferred to PDA slants for long-term storage.

### Screening fungal isolates for mycotoxin producers

The fungal isolates were inoculated into 5 ml of YES broth and incubated at 28 °C for 7 days. After the incubation period, the broth was filtered through Whatman paper into a separating funnel and then extracted with 10 ml chloroform. The extract was then evaporated to dryness and redissolved in 1 ml chloroform (Kumar et al. 2010). Without further purification, the extracts were detected for mycotoxin by TLC (Holcomb et al. 1992; Lin et al. 1998). Ten microliters of the extracts were spotted on a TLC plate along with mycotoxin standards (aflatoxin B_1_, B_2_, G_1_, G_2_, and citrinin) and developed in toluene: ethyl acetate: formic acid (6:3:1, v/v/v) solvent system. The characteristic fluorescence of mycotoxins was visualized under Ultra Violet (UV) light at 365 nm and confirmed chemically by spraying 50 % aqueous sulfuric acid. The Rf values of the standard aflatoxin B_1_, B_2_, G_1_, G_2_, and citrinin were 0.51, 0.47, 0.4, 0.36, and 0.72, respectively, in the solvent system used. The detection limit of aflatoxin B_1_ was noted as 10 ng/ml. The toxigenic fungal isolates were identified on the basis of partial DNA sequence analysis at Agharkar Research Institute, Pune, India.

### Screening herbs for mycotoxin contamination

Five grams of the grounded samples were extracted with 20 ml chloroform. The extract was filtered through Whatman paper, evaporated to dryness, and redissolved in 1 ml chloroform (Hitokoto et al. 1978; Kumar et al. 2010). Analysis by TLC was performed as described above. The chloroform extract was evaporated to dryness and redissolved in benzene for detection of aflatoxin using high-performance liquid chromatography (HPLC) (Waters, Milford, USA). Twenty microliters was injected into the HPLC sampler and water: actenotrile: methanol (60:20:20 v/v/v) was used as mobile phase at a flow rate of 1 ml/min. Aflatoxin was detected with UV detector at a wavelength of 365 nm. The analysis showed the elution of the standard aflatoxin B_1_, B_2_, G_1_, and G_2_ at retention time 9.95, 7.9, 7.19, and 5.77 min, respectively (Supplementary Fig. 1). The performance analysis of HPLC with standard aflatoxin B_1_ showed deviation in retention time and peak area within the acceptable value (<5 %) with a detection limit of 6 ng/ml in the experimental condition used.

## Results

### Fungal contamination and fungal load

In this study, the presence of fungi and mycotoxin in medicinal herbs and spices was examined. The moisture content of all the samples was below 9 %, which is the optimum condition for storage. Table 1 shows the samples that were screened for fungal and mycotoxin contamination. The results obtained showed that out of the 63 samples analyzed, 58 were positive for fungal contamination, while 5 were free from any fungus in the experimental conditions used. *Illicium verum, Plantago ovate*, *Rheum emodi*, *Syzygium aromaticum,* and Herbal drug-3 were the samples that had no fungal contamination. Supplementary Fig. 2 shows the fungal contamination in various herbal samples.

**Table 1:** Fungal contamination in medicinal herbs and spices

S. no.	Sample	Part used	Fungal load (cfu/g)	Isolated fungi	Toxigenic fungi
1	*Acalypha fruticosa* (Chinni)	Leaves	2 × 10^2^	*Aspergillus niger*	–
				*Aspergillus*	–
				*Aspergillus*	–
2	*Acorus calamus* (Sweet flag)	Root	4.5 × 10^2^	*Aspergillus niger*	–
				*Aspergillus*	–
				*Cladosporium*	–
				Unknown	–
3	*Arachis hypogaea* (Groundnut)	Seed	2.75 × 10^3^	*Aspergillus flavus*	AFB_1_
				*Aspergillus niger*	–
				*Aspergillus flavus*	AFB_1_
4	*Asparagus racemosus*-I (Shatawer)	Root	8.5 × 10^3^	*Aspergillus niger*	–
				*Fusarium*	–
				*Penicillium citrinum*	CIT
5	*Asparagus racemosus*-II (Shatawer)	Root	5.0 × 10^2^	*Aspergillus niger*	–
				Unknown	–
6	*Asparagus racemosus*-III (Shatawer)	Root	6.0 × 10^2^	*Aspergillus niger*	–
				Unknown	–
				*Penicillium*	–
				*Penicillium*	–
7	*Asparagus racemosus*-IV (Shatawer)	Root	1.65 × 10^3^	*Aspergillus niger*	–
				*Aspergillus*	–
				*Aspergillus*	–
				*Penicillium*	–
				Unknown	–
8	*Bergenia ciliate* (Winter bergenia)	Root	1.15 × 10^3^	*Aspergillus niger*	–
				*Aspergillus*	–
				*Aspergillus*	–
				*Aspergillus flavus*	AFB_1_
				*Aspergillus*	–
9	*Brassica juncea* (Mustard)	Seed	2.5 × 10^3^	*Penicillium*	–
10	*Chlorophytum borivilianum*-I (Safed musli)	Root	3.5 × 10^3^	*Aspergillus niger*	–
				*Aspergillus flavus*	AFB_1_
				*Aspergillus*	–
				*Fusarium*	–
				*Aspergillus*	–
				*Cladosporium*	–
				*Fusarium*	–
11	*Chlorophytum borivilianum*-II (Safed musli)	Root	8.5 × 10^2^	*Aspergillus*	–
				*Penicillium citrinum*	CIT
				*Penicillium citrinum*	CIT
				*Aspergillus*	–
				*Fusarium*	–
				*Aspergillus*	–
				*Penicillium citrinum*	CIT
				*Aspergillus*	–
12	*Chlorophytum borivilianum*-III (Safed musli)	Root	4.0 × 10^2^	*Aspergillus niger*	–
				*Aspergillus*	–
13	*Chlorophytum borivilianum*-IV (Safed musli)	Root	5.65 × 10^3^	*Aspergillus niger*	–
				*Mucor*	–
14	*Cinnamomum verum* (Cinnamon)	Bark	7.0 × 10^2^	*Aspergillus niger*	–
				*Mucor*	–
15	*Cocus nucifera* (Coconut)	Fruit	5.0 × 10^2^	*Penicillium*	–
16	*Elettaria cardamomum* (Cardamom)	Seed pod	7.5 × 10^2^	*Aspergillus niger*	–
				*Mucor*	–
				*Rhizopus*	–
17	*Ficus arnottiana*-I (Paras peepal)	Bark	2.2 × 10^3^	*Aspergillus niger*	–
				*Penicillium citrinum*	CIT
				*Penicillium*	–
18	*Ficus arnottiana*-II (Paras peepal)	Bark	2.6 × 10^3^	*Penicillium citrinum*	CIT
				*Aspergillus*	–
19	*Ficus arnottiana*-I (Paras peepal)	Flower	3.0 × 10^2^	*Aspergillus niger*	–
				*Mucor*	–
20	*Ficus arnottiana*-II (Paras peepal)	Flower	3.5 × 10^2^	*Aspergillus niger*	–
				Unknown	–
				*Mucor*	–
21	*Ficus arnottiana*-III (Paras peepal)	Flower	5.5 × 10^2^	*Aspergillus niger*	–
				*Penicillium*	–
				Unknown	–
				*Aspergillus*	–
22	*Ficus arnottiana*-IV (Paras peepal)	Flower	10.4 × 10^3^	*Aspergillus niger*	–
				*Mucor*	–
				*Penicillium*	–
23	*Foeniculum vulgare*-I (Fennel)	Seed	3.5 × 10^2^	*Alternaria*	–
				*Mucor*	–
				*Aspergillus*	–
24	*Foeniculum vulgare*-II (Fennel)	Seed	2.0 × 10^3^	Unknown	–
25	*Glycyrrhiza glabra*-I (Mulethi)	Root	1.75 × 10^3^	*Aspergillus*	–
				*Penicillium*	–
				*Aspergillus*	–
				*Cladosporium*	–
				*Penicillium citrinum*	CIT
26	*Glycyrrhiza glabra*-II (Mulethi)	Root	3.2 × 10^3^	*Aspergillus niger*	–
				*Penicillium*	–
				*Penicillium*	–
				*Penicillium*	–
				*Aspergillus flavus*	AFB_1_
				*Alternaria*	–
				*Penicillium*	–
27	*Glycyrrhiza glabra*-III (Mulethi)	Root	5.0 × 10^3^	*Aspergillus niger*	–
				*Aspergillus flavus*	AFB_1_
				*Penicillium*	–
				*Aspergillus*	–
				Unknown	–
28	Herbal drug-1	Unknown herb mixture	5.0 × 10^1^	*Aspergillus flavus*	AFB_1_
29	Herbal drug-2	Unknown herb mixture	1.5 × 10^4^	*Aspergillus Niger*	–
				*Aspergillus*	–
				*Aspergillus*	–
				*Aspergillus*	–
30	Herbal drug-3	Unknown herb mixture	ND	ND	–
31	Herbal drug-4	Unknown herb mixture	3.5 × 10^2^	*Penicillium citrinum*	CIT
				*Aspergillus*	–
32	Herbal drug-5	Unknown herb mixture	2.0 × 10^2^	*Aspergillus niger*	–
				*Mucor*	–
				*Aspergillus*	–
33	Herbal drug-6	Unknown herb mixture	2.3 × 10^3^	*Aspergillus*	–
				*Aspergillus*	–
				*Aspergillus*	–
				*Aspergillus*	–
34	Herbal drug-7	Unknown herb mixture	2.0 × 10^2^	*Penicillium*	–
				*Penicillium*	–
35	*Illicium verum* (Star anise)	Fruit	ND	ND	–
36	*Mesua ferrea*-I (Nagkeshar)	Flower bud	7.5 × 10^2^	*Penicillium citrinum*	CIT
				*Penicillium*	–
				*Aspergillus*	–
				*Aspergillus*	–
				*Aspergillus flavus*	AFB_1_
				*Aspergillus*	–
37	*Mesua ferrea*-II (Nagkeshar)	Flower bud	5.0 × 10^4^	*Aspergillus niger*	–
				*Aspergillus flavus*	AFB_1_
				*Aspergillus flavus*	AFB_1_
38	*Mesua ferrea*-III (Nagkeshar)	Flower bud	2.6 × 10^3^	*Aspergillus niger*	–
				*Mucor*	–
				*Aspergillus flavus*	AFB_1_
				*Penicillium*	–
				*Cladosporium*	–
39	*Myristica fragrans* (Mace)	Seed	6.5 × 10^2^	*Aspergillus niger*	–
				*Mucor*	–
				*Aspergillus*	–
40	Nimbapatradi churnam	Unknown herb mixture	8.5 × 10^2^	*Aspergillus flavus*	AFB_1_
				*Aspergillus*	–
				*Aspergillus*	–
41	*Oroxylum indicum* (Indian trumpet)	Stem bark	1.8 × 10^3^	*Aspergillus*	–
				*Aspergillus*	–
				*Aspergillus*	–
				*Aspergillus*	–
				*Cladosporium*	–
				*Fusarium*	–
				*Aspergillus niger*	–
42	*Phyllanthus emblica*-I (Amla)	Fruit	6.0 × 10^2^	*Aspergillus*	–
				*Cladosporium*	–
43	*Phyllanthus emblica*-II (Amla)	Fruit	9.0 × 10^2^	*Aspergillus niger*	–
				*Cladosporium*	–
				*Aspergillus*	–
				Unknown	–
44	*Phyllanthus emblica*-III (Amla)	Fruit	1.75 × 10^3^	*Aspergillus niger*	–
				*Penicillium*	–
				*Aspergillus*	–
				*Aspergillus*	–
				*Cladosporium*	–
45	*Piper longum* (Long pepper)	Fruit	4.0 × 10^2^	*Mucor*	–
46	*Piper nigrum* (Pepper)	Fruit	1.0 × 10^2^	*Aspergillus niger*	–
				*Cladosporium*	–
47	*Plantago ovate* (Isabgol)	Fruit	ND	ND	–
48	*Rheum emodi* (Himalayan rubhada)	Root	ND	ND	–
49	*Sesamum indicum* (Sesame seed)	Seed	7.0 × 10^2^	*Aspergillus niger*	–
				*Aspergillus niger*	–
				*Aspergillus flavus*	AFB_1_
				*Aspergillus flavus*	AFB_1_
50	*Stevia rebaudiana* (Stevia)	Leaves	3.5 × 10^2^	*Aspergillus*	–
				*Fusarium*	–
				*Alternaria*	–
51	*Syzygium aromaticum* (Clove)	Flower bud	ND	ND	–
52	*Terminalia belerica*-I (Baheda)	Fruit	2.0 × 10^3^	*Aspergillus niger*	–
53	*Terminalia belerica*-II (Baheda)	Fruit	2.0 × 10^3^	*Aspergillus niger*	–
				*Mucor*	–
54	*Terminalia belerica*-III (Baheda)	Fruit	2.5 × 10^4^	*Aspergillus niger*	–
				*Aspergillus flavus*	AFB_1_
				*Aspergillus flavus*	AFB_1_
55	*Terminalia chebula*-I (Harre badi)	Fruit	3.0 × 10^3^	*Aspergillus niger*	–
				*Aspergillus niger*	–
				*Cladosporium*	–
				*Penicillium*	–
				*Aspergillus*	–
				*Aspergillus flavus*	AFB_1_
56	*Terminalia chebula*-II (Harre badi)	Fruit	8.0 × 10^3^	*Aspergillus niger*	–
57	*Terminalia chebula*-III (Harre badi)	Fruit	5.0 × 10^4^	*Aspergillus niger*	–
58	*Terminalia chebula*-I (Harre choti)	Fruit	4.5 × 10^2^	*Aspergillus niger*	–
59	*Terminalia chebula*-II (Harre choti)	Fruit	8.0 × 10^2^	*Aspergillus niger*	–
				*Aspergillus flavus*	AFB_1_
				*Aspergillus*	–
60	*Terminalia chebula*-III (Harre choti)	Fruit	5.0 × 10^4^	*Aspergillus niger*	–
61	*Withania somnifera*-I (Ashwagandha)	Root	2.6 × 10^3^	*Mucor*	–
				*Aspergillus niger*	–
62	*Withania somnifera*-II (Ashwagandha)	Root	3.2 × 10^3^	*Aspergillus niger*	–
				*Aspergillus flavus*	AFB_1_
				*Mucor*	–
63	*Withania somnifera*-III (Ashwagandha)	Root	5.5 × 10^2^	*Aspergillus niger*	–
				*Mucor*	–
				*Penicillium*	–
				Unknown	–
				*Cladosporium*	–
				Unknown	–

Samples collected at different intervals are represented as I, II, III, and IV

*AFB*_*1*_ aflatoxin B_1_, *CIT* citrinin, *ND* none detected in the experimental conditions used

The fungal load in the samples were enumerated as colony forming unit per gram (CFU/g) of sample and given in Table 1. Nine herbs comprising 14.28 % of the total samples had the highest fungal contamination (5.0 × 10^3^ to 5.0 × 10^4^ cfu/g) which includes *Asparagus racemosus*-*I*, *Chlorophytum borivilianum*-*IV*, *Ficus arnottiana*-*IV*, Herbal drug-2, *Mesua ferrea*-*II*, *Terminalia belerica*-*III,* *Terminalia chebula*-*II (Harre badi)*, *Terminalia chebula*-*III (Harre badi)*, and *Terminalia chebula*-*III (Harre choti)*. The majority of the herbs (52 %) had a fungal load in the range of 5.0 × 10^2^ to 5.0 × 10^3^. Samples that contain the least fungal load (5.0 × 10^1^ to 3.5 × 10^2^ cfu/g) include *Asparagus racemosus*-2, Herbal drug-1, *Ficus arnottiana*-1 (Fruit), *Ficus arnottiana*-2 (Fruit), Herbal drug-5, Herbal drug-7, and *Acalypha fruticosa*. The result showed that 45.31 % of the samples have a fungal load above the permissible limit of the World Health Organization (WHO 2005). A contamination limit of 1 × 10^3^ cfu/g has been set by WHO for yeasts and molds in medicinal plants.

### Fungal isolates

A total of 187 fungi were isolated from the samples of medicinal herbs, spices, and food materials (Table 1). The different genus of the fungi, such as *Aspergillus*, *Penicillium*, *Mucor*, *Cladosporium*, *Fusarium*, *Alternaria,* and *Rhizopus,* were identified. Among the various species of *Aspergillus*, *Aspergillus niger* was found to be the most commonly occurring contaminant and was isolated from 42 (66.66 %) samples. The fungal isolates were further screened for mycotoxin producers especially for aflatoxins and citrinin (Supplementary Fig. 3). Twenty-eight fungi out of 187 isolates were mycotoxin producers which included 19 aflatoxin B_1_-producing *Aspergillus flavus* and 9 citrinin-producing *Penicillium citrinum*. The toxins were further confirmed by running the extracts spiked with aflatoxins and citrinin standards on TLC plates and spraying with aqueous sulphuric acid. The toxigenic *Aspergillus* isolates were identified as *Aspergillus flavus* Link 1809 similarity with NCBI sequence accession HQ14704.1, whereas the toxigenic *Penicillium* isolates were identified as *Penicillium citrinum* Sopp 1910 similarity with NCBI sequence accession HQ232482.1.

Toxigenic fungi were isolated from samples that had low fungal load, such as *Sesamum indicum* and Herbal drug-1; while samples that had a high fungal load, such as *Terminalia chebula*-4 and Herbal drug-2, had no toxigenic fungi. Aflatoxin B_1_-producing *A. flavus* and citrinin-producing *P. citrinum* were isolated from all the samples of *Glycyrrhiza glabra*. A total of 19 fungi were isolated from the four samples of *Chlorophytum borivilianum* out of which three were *P. citrinum* producing citrinin and one was *A. flavus* producing aflatoxin B_1_. When the bark and fruit of *Ficus arnottiana* were screened, the occurrence of citrinin-producing *Penicillium* on the bark and the absence of toxigenic fungi on the fruit were found to be consistent in all the samples collected at different time periods. The presence of toxigenic *A. flavus* on *Mesua ferrea* occurred consistently when screened again after a period of 6 and 12 months. All the samples of *Asparagus racemosus* which is commonly used in herbal drugs were contaminated with fungi; however, none of the *Aspergillus* isolates were toxigenic, and only one *P. citrinum* was a citrinin producer.

### Mycotoxin contamination

About 33 % of the samples analyzed showed the presence of toxigenic fungi producing aflatoxins and citrinin, which indicate the potential for the presence of mycotoxins in herbs. Therefore, the herbs were further screened for natural contamination with aflatoxins and citrinin. Of the 63 samples analyzed, only one sample was found to be contaminated with aflatoxin B_1_. The HPLC analysis of *Arachis hypogaea* (groundnut) sample showed the presence of a peak at a retention time of 10.06 min which corresponded with the standard aflatoxin B_1_ (Supplementary Fig. 4). The concentration of aflatoxin B_1_ in groundnut was found to be 2.5 mg/kg, which is beyond the Indian permissible limit, i.e., 30 µg/kg. This groundnut sample was highly contaminated with toxigenic *Aspergillus*. Though toxigenic fungi were present in the other samples, mycotoxins were not detected from any of the herbs and medicinal plants. The result suggests that the herbs either were free from mycotoxin contamination or present below the detectable limit.

## Discussion

Mycotoxins have been identified as important toxins affecting animal species and humans ever since the discovery of aflatoxin B_1_ in 1960. Mycotoxigenic fungi are ubiquitous in nature and are held responsible for economic loss by decreasing the crop yield and quality of food. The presence of fungi and their mycotoxins are reported not only in food grains but also in medicinal herbs and processed foods. In this study, medicinal herbs and spices were screened for the presence of fungi and mycotoxin. Among the various fungal isolates, *Aspergillus* and *Penicillium* were the predominant fungi isolated which corresponded with previous reports. Species of *Aspergillus, Penicillium*, and *Fusarium* are the major producers of mycotoxins, and hence, there occurrence in the samples is of high significance. Efuntoye (1996) had also reported that species of the genera *Aspergillus, Penicillium*, and *Fusarium* were the most abundant fungi present in medicinal herbs. Examination of 84 medicinal plants and spices revealed *A. flavus*, *A. parasiticus*, *F. oxysporum*, and *P. viridicatum* as the most commonly occurring contaminant (Aziz et al. 1998). Other genera, such as *Fusarium, Mucor* and *Trichoderma*, were also reported as dominant fungi present in medicinal plants and herbal drugs (Efuntoye 1996; Martins et al. 2001). *Aspergillus niger* has been reported as a frequent contaminant in medicinal plants (Efuntoye 1996; Abou-Arab et al. 1999; Bugno et al. 2006). The presence of *A. niger* on the herbs may or may not cause any ill effects.

Although *Aspergillus*, *Penicillium*, and *Fusarium* are the major producers of mycotoxins, not all species are toxigenic. As seen from this study, though all the samples of *Phyllanthus emblica* were contaminated with several species of *Aspergillus*, *Cladosporium*, and *Penicillium*, none of the isolates were found toxigenic. It was reported that only 21.9 % of *Aspergillus* and *Penicillium* isolates from herbal drugs were producers of aflatoxins, ochratoxin A, and citrinin (Bugno et al. 2006). Similarly, Aziz et al. (1998) had reported that 37 % of *Aspergillus* isolates from medicinal plants and spices were aflatoxin producers.

Groundnuts are known to be commonly contaminated with aflatoxin B_1_, and in this study, a high amount of aflatoxin B_1_ on groundnut sample was detected. It is interesting to note that none of the medicinal herbs and spices was positive for the toxins analyzed. The samples from which toxigenic fungi were isolated also tested negative for natural mycotoxin contamination. An earlier investigation by Abou-Arab et al. (1999) reported the occurrence of *Aspergillus*, *Penicillium*, and *Fusarium* species in medicinal plants; however, natural mycotoxin contamination was absent in all the samples. Another investigator reported the presence of aflatoxins in spices but interestingly not in aromatic herb, herb-tea, and medicinal plant samples (Romagnoli et al. 2007). In conformity with the findings of earlier investigators, this study also suggests that the presence of toxigenic fungi does not mean mycotoxin contamination in herbs.

Though several toxigenic strains were isolated from the samples, the herb itself may not be a good substrate for toxin production. Many fungal strains which produce toxins in synthetic medium were unable to produce toxins in medicinal plants. The absence of mycotoxins as a natural contaminant in samples may be due to intrinsic characteristics of herbs such as essential oils which inhibit the toxin production (Al-Rahmah et al. 2011; Prakash et al. 2011). The herb *Withania somnifera* has been reported to possess the ability to inhibit aflatoxin B_1_ synthesis (Krishnamurthy and Shashikala 2006). In addition, in this study, aflatoxin-producing *Aspergillus flavus* was isolated from this herb; however, aflatoxin B_1_ itself was not detected. The antioxidant property of several plants, especially herbs and spices, is detrimental to secondary metabolism and, hence, has the natural potential for inhibiting mycotoxin. It has been demonstrated that the presence of active oxygen is favorable for aflatoxin B_1_ production by Narasaiah et al. (2006). Hence, the antioxidants of several plants have been extensively studied as anti-fungal and anti-mycotoxin agents (Gulcin et al. 2012; Mahoney et al. 2010). The powder and essential oil of *Cymbopogon citratus* have been shown to inhibit aflatoxin B_1_ and also preserve the quality of melon seed under storage (Bankole and Joda 2004). The essential oils of *Cinnamomum jensenianum* (Tian et al. 2011), *Ocimum sanctum* (Kumar et al. 2010), and *Zataria multiflora* (Gandomi et al. 2009) were efficiently used against toxigenic fungi and aflatoxin B_1_, and their safe use as natural preservative of food has been implicated. The use of whole spices, such as *Syzygium aromaticum* and *Cinnamomum verum*, has been shown to inhibit the growth of *A. flavus* and *P. citrinum* and their toxins in culture media and rice grains (Aiko and Mehta 2013a, b). These reports corroborated with the observations of the present study in which the absence of mycotoxin could be attributed to the inhibitory property of the herbs and spices.

As the environmental conditions are conducive for the fungal growth in tropical region, most of the samples investigated in this study were heavily contaminated with toxigenic as well as non-toxigenic fungi. The sample *Asparagus racemosus* collected from the herbal farm showed heavy contamination, while the same sample in packed form was less contaminated. Thus, suggesting that proper cleaning and packaging is effective in reducing the fungal contamination. However, several other herbal drugs that were packed and sold in the market, such as Nimbapatradi churnam, Harre badi, Safed museli, Herbal drug-1, and Herbal drug-4, had high fungal load, and toxigenic fungi were present in each of them. Though mycotoxin was not detected from these herbs, the presence of toxigenic fungi poses a risk of mycotoxin contamination and, thereby, subject humans to potential health threats.

## Conclusion

Medicinal plants and herbs are commonly used in most households in the form of culinary for flavor and aroma, as herbal teas or to alleviate illness. This study concludes that medicinal herbs and spices can be contaminated with mycotoxins-producing fungi. The results showed that not only the raw materials but the processed herbs as well are susceptible to toxigenic fungi. Contamination of these products with toxigenic fungi poses serious health threats, since their presence can cause ill effects rather than improving the quality of life. Proper storage conditions and quality control at every stage of processing, packaging or marketing are necessary for the safety of the consumers. This study provides a basis in assessing the degree of fungal and potential mycotoxin contamination in medicinal plants, herbs, and spices, thus providing quality control.

## Electronic supplementary material

Below is the link to the electronic supplementary material.

**Supplementary Fig.1** HPLC analysis of standard aflatoxin B_1_, B_2_, G_1_, and G_2_ at a retention time of 9.95, 7.9, 7.16, and 5.77 min, respectively, detected at 365 nm (JPEG 1019 kb).

**Supplementary Fig. 2** RBCA plates showing fungal growth from herbal samples. 1-*Chlorophytum borivilianum*, 2-*Phyllanthus emblica-*II, 3- *Sesamum indicum*, 4-*Terminalia chebula*, 5- *Arachis hypogaea*, 6-*Acorus calamus*, 7-*Oroxylum indicum*, 8-*Glycyrrhiza glabra*, 9-*Brassica juncea*, 10-Herbal drug-6, 11- *Ficus arnottiana* (fruit), 12- *Asparagus racemosus*, 13-*Phyllanthus emblica*-I, 14- *Bergenia ciliata*, 15-*Mesua ferrea*, 16- *Chlorophytum borivilianum*-3, 17-*Withania somnifera*, 18-Herbal drug-5 (JPEG 2170 kb).

**Supplementary Fig. 3** TLC plate showing Citrinin as yellow streaks (Rf- 0.72) and Aflatoxin as blue spots (Rf- B_1_ 0.51, B_2_ 0.47, G_1_ 0.4, G_2_ 0.36) extracted from the fungal isolates. Lane 1-Citrinin standard, 2-*Ficus arnottiana*-I, 3-*Mesua ferrea*, 6-*Chlorophytum borivilianum*, 9-*Glycyrrhiza glabra*-II, 11-*Ficus arnottiana*-II, 12-*Glycyrrhiza glabra*-I, 15-*Withania somnifera*-II, 20-Aflatoxin standard, 22-*Asparagus racemosus-*I, 24-*Sesamum indicum*, 26-*Arachis hypogaea*, 28-*Bergenia ciliate* (JPEG 419 kb).

**Supplementary Fig.4** HPLC analysis of *Arachis hypogaea* (groundnut) sample showing aflatoxin B_1_ peak at 10.06 min retention time (JPEG 1027 kb)

## References

[CR1] Abou-Arab AAK, Kawther MS, El Tantawy ME, Badeae RI, Khayrai N (1999). Quality estimation of some contaminants in commonly used medicinal plants in the Egyptian market. Food Chem.

[CR2] Aiko V, Mehta A (2013). Control of *Aspergillus flavus* and aflatoxin production with spices in culture media and rice. World Mycotoxin J.

[CR3] Aiko V, Mehta A (2013). Inhibitory effect of clove (*Syzygium aromaticum*) on the growth of *Penicillium citrinum* and citrinin production. J Food Saf.

[CR4] Al-Rahmah N, Mostafa A, Abdel-Megeed A (2011). Antifungal and antiaflatoxigenic activities of some plant extracts. Afr J Microbiol Res.

[CR5] Aziz NH, Youssef YA, El-Fouly MZ, Moussa LA (1998). Contamination of some common medicinal plant samples and spices by fungi and their mycotoxins. Bot Bull Acad Sin.

[CR6] Bankole SA, Joda AO (2004). Effect of lemon grass (*Cymbopogon citratus* Stapf) powder and essential oil on mould deterioration and aflatoxin contamination of melon seeds (*Colocynthis citrullus* L.). Afr J Biotechnol.

[CR7] Bokhari FM (2007). Spices mycobiota and mycotoxins available in Saudi Arabia and their abilities to inhibit growth of some toxigenic fungi. Mycobiology.

[CR8] Bugno A, Almodovar AAB, Pereira TC, Pinto TJA, Sabino M (2006). Occurrence of toxigenic fungi in herbal drugs. Braz J Microbiol.

[CR9] Calixto JB (2000). Efficacy, safety, quality control, marketing and regulatory guidelines for herbal medicines (phytotherapeutic agents). Braz J Med Biol Res.

[CR10] Cho SH, Lee CH, Jang MR, Son YW, Lee SM, Choi IS, Kim SM, Kim DB (2008). Aflatoxin contamination in spices and processed spice products commercialized in Korea. Food Chem.

[CR11] Chourasia HK (1995). Mycobiota and mycotoxins in herbal drugs of Indian pharmaceutical industries. Mycol Res.

[CR12] Efuntoye MO (1996). Fungi associated with herbal drug plants during storage. Mycopathologia.

[CR13] Gandomi H, Misaghi A, Basti AA, Bokaei S, Khosravi A, Abbasifar A, Javan AJ (2009). Effect of *Zataria multiflora* Boiss. Essential oil on growth and aflatoxin formation by *Aspergillus flavus* in culture media and cheese. Food Chem Toxicol.

[CR14] Gulcin I, Elmastas H, Aboul-Enein HY (2012). Antioxidant activity of clove oil—a powerful antioxidant. Arabian J Chem.

[CR15] Hitokoto H, Morozumi S, Wauke T, Sakai S, Kurata H (1978). Fungal contamination and mycotoxin detection in powdered herbal drugs. Appl Environ Microbiol.

[CR16] Hocking AD, Pitt JI, Samson RA, Thrane U (2006) Advances in food mycology. Springer Publishers 571(4):343–348

[CR17] Holcomb M, Wilson DM, Trucksess MW, Thomson HC (1992). Determination of aflatoxins in food products by chromatography. J Chromatogr A.

[CR18] Hussain SH, Brasel JM (2001). Toxicity, metabolism and impact of mycotoxins on humans and animals. Toxicology.

[CR19] Krishnamurthy YL, Shashikala J (2006). Inhibition of aflatoxin B1 production of *Aspergillus flavus*, isolated from soybean seeds by certain natural plant products. Lett Appl Microbiol.

[CR20] Kumar A, Shukla R, Singh P, Dubey NK (2010). Chemical composition, antifungal and antiaflatoxigenic activities of *Ocimum sanctum* L. essential oil and its safety assessment as plant based antimicrobial. Food Chem Toxicol.

[CR21] Lin L, Zhang J, Wang P, Wang Y, Chen J (1998). Thin-layer chromatography of mycotoxins and comparison with other chromatographic methods. J Chromatogr A.

[CR22] Mahoney NE, Molyneux R, Kim JH, Campbell BC, Waiss AC, Hagerman AE (2010). Aflatoxigenesis induced in *Aspergillus flavus* by oxidative stress and reduction by phenolic antioxidants from tree nuts. World Mycotoxin J..

[CR23] Martins HM, Martins ML, Dias MI, Bernardo F (2001). Evaluation of microbiological quality of medicinal plants used in natural infusions. Int J Food Microbiol.

[CR24] Narasaiah KV, Sashidhar RB, Subramanyam C (2006). Biochemical analysis of oxidative stress in the production of aflatoxin and its precursor intermediates. Mycopathologia.

[CR25] Nielsen SS (2010) Food analysis laboratory manual. Food science text series. Springer Publishers 17

[CR26] Prakash B, Shukla R, Singh P, Mishra PK, Dubey NK, Kharwar RN (2011). Efficacy of chemically characterized *Ocimum gratissimum* L. essential oil as an antioxidant and a safe plant based antimicrobial against fungal and aflatoxin B1 contamination of spices. Food Res Int.

[CR27] Rizzo I, Vedoya G, Maurutto S, Haidukowski M, Varsavsky E (2004). Assessment of toxigenic fungi on Argentinean medicinal herbs. Microbiol Res.

[CR28] Romagnoli B, Menna V, Gruppioni N, Bergamini C (2007). Aflatoxins in spices, aromatic herbs, herb-teas and medicinal plants marketed in Italy. Food Control.

[CR29] Roy AK, Sinha KK, Chourasia HK (1988). Aflatoxin contamination of some common drug plants. Appl Environ Microbiol.

[CR30] Santos L, Marin S, Sanchis V, Ramos AJ (2009). Screening of mycotoxin multicontamination in medicinal and aromatic herbs sampled in Spain. J Sci Food Agr.

[CR31] Sewram V, Shephard GS, Merwe LVD, Jacobs TV (2006). Mycotoxin contamination of dietary and medicinal wild plants in the Eastern Cape Province of South Africa. J Agr Food Chem.

[CR32] Thirumala-Devi K, Mayo MA, Reddy G, Emmanuel KE, Larondelle Y, Reddy DVR (2001). Occurrence of ochratoxin A in black pepper, coriander, ginger and turmeric in India. Food Addit Contam.

[CR33] Tian J, Huang B, Luo Z, Zeng H, Ban X, He J, Wang Y (2011). The control of *Aspergillus flavus* with *Cinnamomum jensenianum* Hand.-Mazz essential oil and its potential use as a food preservative. Food Chem.

[CR34] Trucksess MW, Scott PM (2008). Mycotoxins in botanical and dried fruits: a review. Food Addit Contam.

[CR35] Viswanathan MV, Singh HB, Bhagwat PR (2000). Handbook on Herbaria in India and Neighbouring Countries.

[CR36] World Health Organisation (2005) Quality control methods for medicinal plant materials. World Health Organization. Working document QAS/05.131/Rev.1, 37

