# Supplementary figures and images for: Prevalence of toxigenic fungi in common medicinal herbs and spices in India
Source: 3 Biotech. 2016 Aug 5;6(2):159. doi: 10.1007/s13205-016-0476-9 (PMC4975724; doi:10.1007/s13205-016-0476-9)

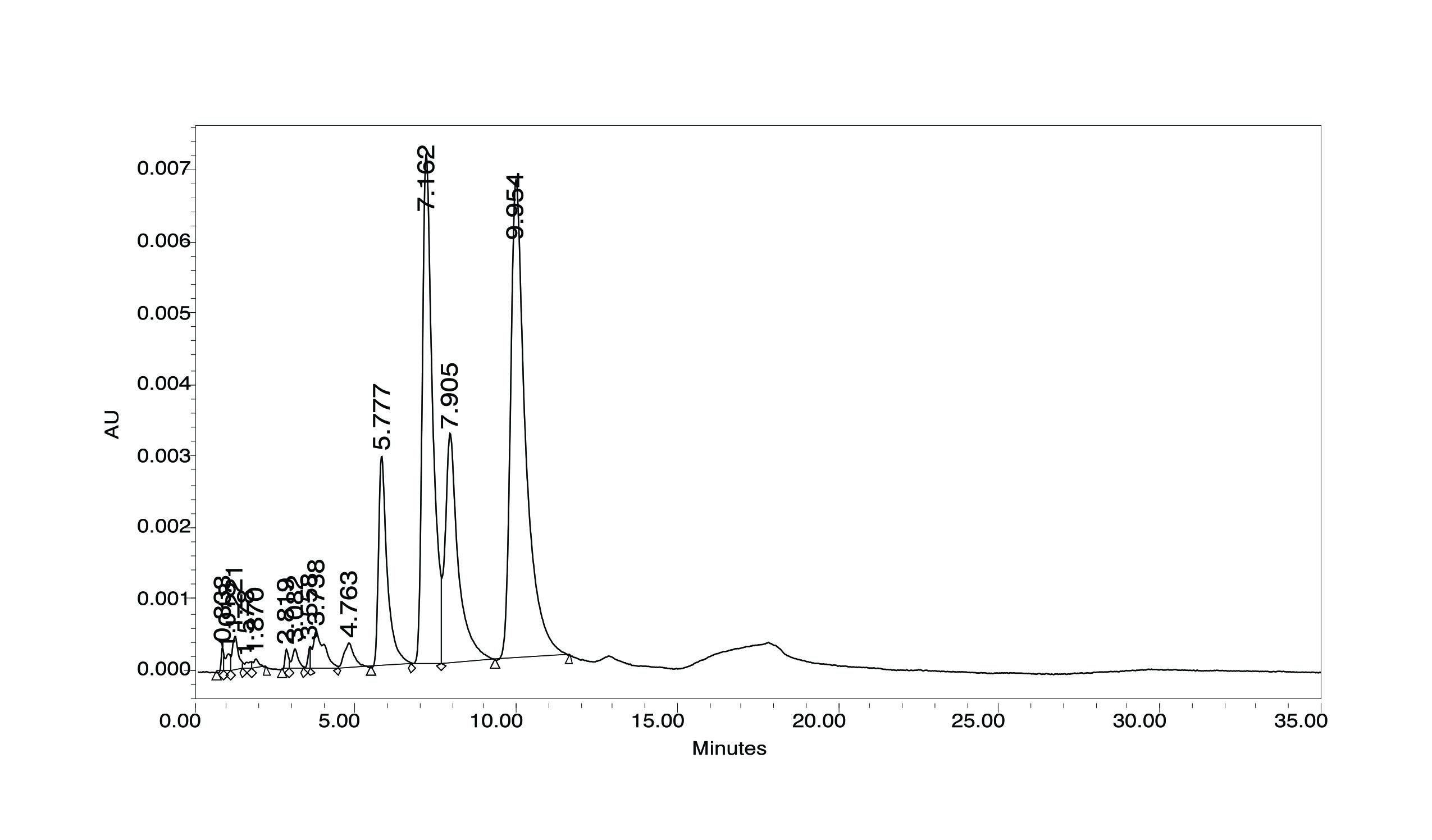

Supplement: Supplementary file 1 — Supplementary Fig.1 HPLC analysis of standard aflatoxin B1, B2, G1, and G2 at a retention time of 9.95, 7.9, 7.16, and 5.77 min, respectively, detected at 365 nm (JPEG 1019 kb). [file 13205_2016_476_MOESM1_ESM.jpg]

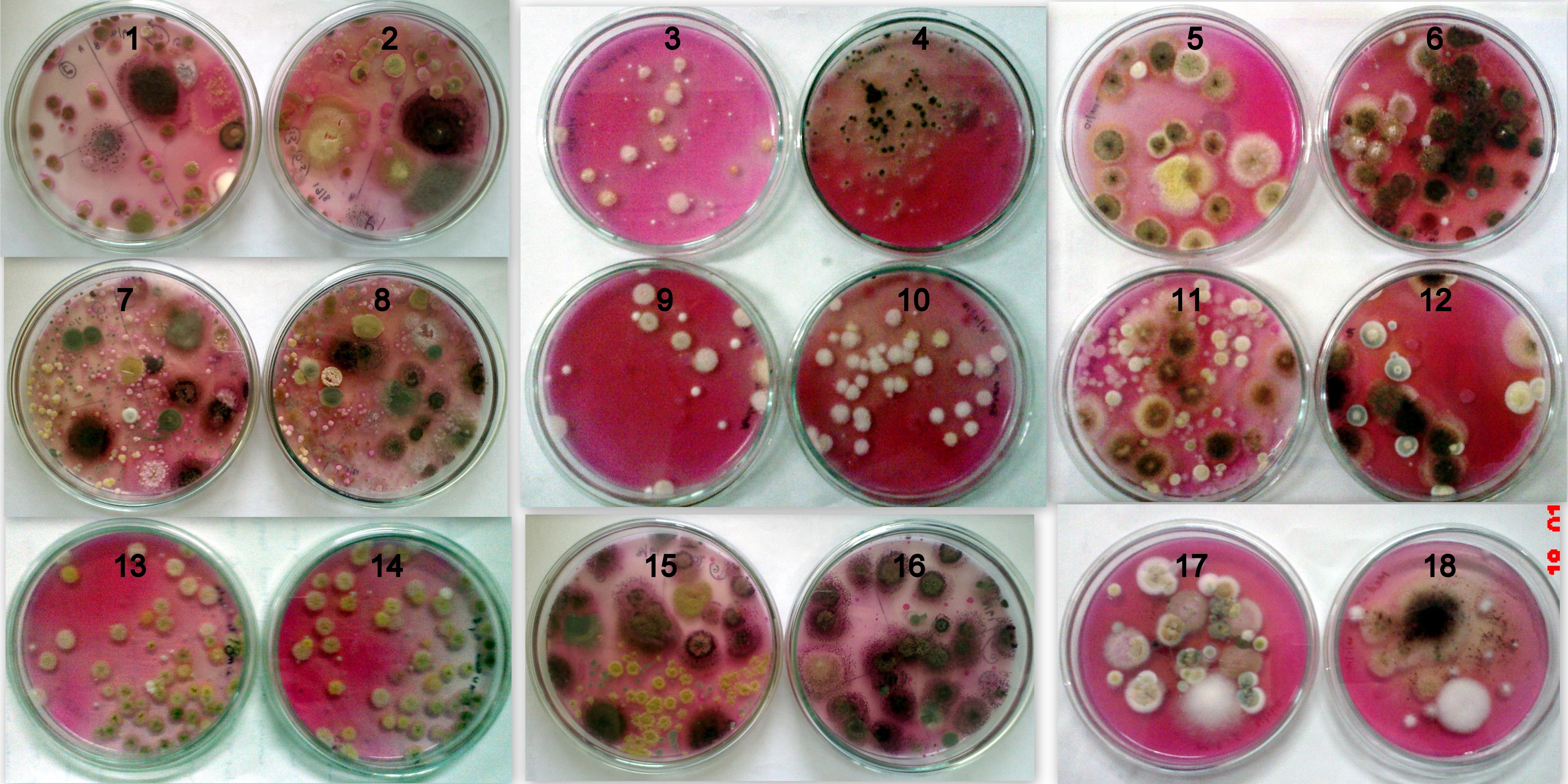

Supplement: Supplementary file 2 — Supplementary Fig. 2 RBCA plates showing fungal growth from herbal samples. 1-Chlorophytum borivilianum, 2-Phyllanthus emblica-II, 3- Sesamum indicum, 4-Terminalia chebula, 5- Arachis hypogaea, 6-Acorus calamus, 7-Oroxylum indicum, 8-Glycyrrhiza glabra, 9-Brassica juncea, 10-Herbal drug-6, 11- Ficus arnottiana (fruit), 12- Asparagus racemosus, 13-Phyllanthus emblica-I, 14- Bergenia ciliata, 15-Mesua ferrea, 16- Chlorophytum borivilianum-3, 17-Withania somnifera, 18-Herbal drug-5 (JPEG 2170 kb). [file 13205_2016_476_MOESM2_ESM.jpg]

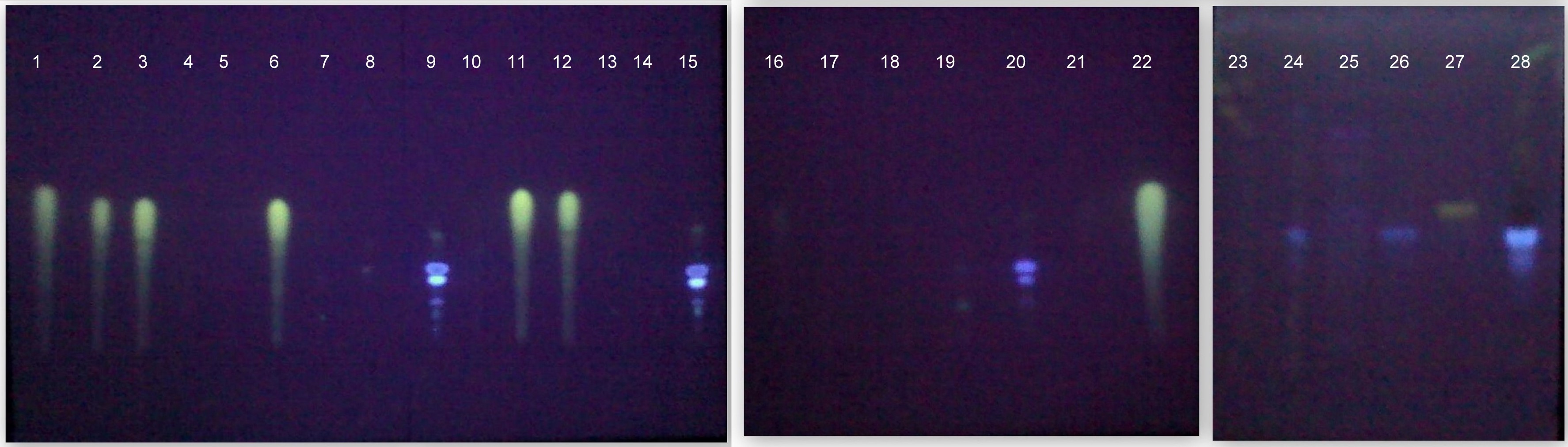

Supplement: Supplementary file 3 — Supplementary Fig. 3 TLC plate showing Citrinin as yellow streaks (Rf- 0.72) and Aflatoxin as blue spots (Rf- B1 0.51, B2 0.47, G1 0.4, G2 0.36) extracted from the fungal isolates. Lane 1-Citrinin standard, 2-Ficus arnottiana-I, 3-Mesua ferrea, 6-Chlorophytum borivilianum, 9-Glycyrrhiza glabra-II, 11-Ficus arnottiana-II, 12-Glycyrrhiza glabra-I, 15-Withania somnifera-II, 20-Aflatoxin standard, 22-Asparagus racemosus-I, 24-Sesamum indicum, 26-Arachis hypogaea, 28-Bergenia ciliate (JPEG 419 kb). [file 13205_2016_476_MOESM3_ESM.jpg]

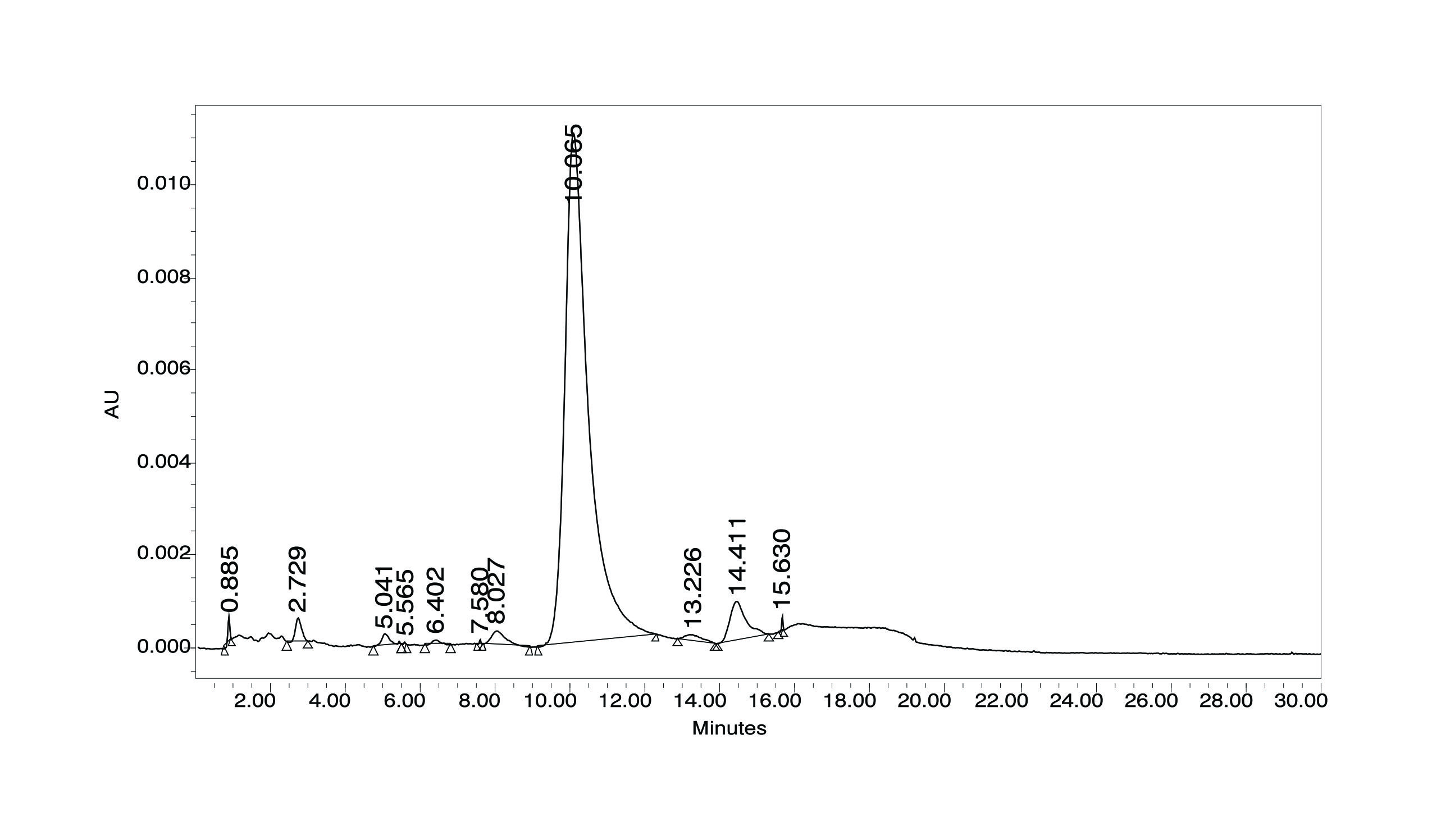

Supplement: Supplementary file 4 — Supplementary Fig.4 HPLC analysis of Arachis hypogaea (groundnut) sample showing aflatoxin B1 peak at 10.06 min retention time (JPEG 1027 kb) [file 13205_2016_476_MOESM4_ESM.jpg]
